# Comparison of cardiovascular risk assessment protocols and achievement of risk factor targets in patients with type 2 diabetes

**DOI:** 10.3389/fendo.2025.1572202

**Published:** 2025-07-03

**Authors:** Hui Guo, Huizhen Wu

**Affiliations:** ^1^ Graduate School of Hebei Medical University, Shijiazhuang, China; ^2^ Hebei Key Laboratory of Clinical Pharmacy, Department of Pharmacy, Hebei General Hospital, Shijiazhuang, China; ^3^ Department of Pharmacy, The Second Affiliated Hospital of Hebei North University, Zhangjiakou, China

**Keywords:** type 2 diabetes, 10-year cardiovascular risk, SCORE2-Diabetes model, cardiovascular risk assessment model, cardiovascular disease

## Abstract

**Objective:**

Compare the differences in the 10-year cardiovascular risk assessment results for patients with type 2 diabetes among various guidelines, as well as the attainment of target levels for primary cardiovascular risk factors.

**Method:**

This study is a retrospective, real-world study that included patients with type 2 diabetes who were hospitalized at the Second Affiliated Hospital of Hebei North University from August 2023 until October 2023.This study evaluated the 10-year cardiovascular risk in patients with type 2 diabetes, as well as the attainment of target levels for blood pressure, blood glucose, and lipid levels.

**Result:**

This study included a total of 200 hospitalized patients with type 2 diabetes, with a median age of 62.5 (57, 67), among which 97 (48.5%)were male. According to the SCORE2-Diabetes model from the European Society of Cardiology’s “2023 ESC Guidelines for the management of cardiovascular disease in patients with diabetes”, the assessment results were as follows:165 cases(82.5%) were classified as very high risk,25 cases(10%) as high risk,8 cases(4%) as medium risk, and 2 cases(1%) as low risk; According to the assessment method from the American Association of Clinical Endocrinologists’ “Comprehensive Management Algorithm for Type 2 Diabetes(2023 edition)”, the assessment results were as follows:150 cases(75%) were classified as extreme risk, 48 cases(24%) as very high risk, and 2 cases(1%) as high risk; The assessment results using the method outlined in the “Guideline for the Prevention and Treatment of Diabetes Mellitus in China(2024 Edition)”by the Chinese Diabetes Society are as follows:149 cases(74.5%) are at extremely high risk, and 51 cases(25.5%) are at high risk. There is no statistically significant difference among the assessment results of the three methods(χ²=2.759, P=0.252).Based on the criteria in the aforementioned three guidelines, the achievement rates for the main cardiovascular risk factors were assessed, with rates of 1%, 0%, and 0% respectively. There is no statistically significant difference among these results(P=0.332).

**Conclusion:**

Although the methods for assessing 10-year cardiovascular risk and the criteria for achieving main cardiovascular risk factor targets differ among the three guidelines, there is no statistical difference in the assessment results and achievement rates among the 200 patients with type 2 diabetes.

## Introduction

Over the past three decades, the prevalence of diabetes has risen significantly, with the number of adults aged 18 and over with diabetes surging from approximately 200 million in 1990 to 828 million in 2022 globally, among them, the number of adult diabetes patients in China is about 148 million, accounting for 18% of the global total and ranking second (1). According to “China Cardiovascular Health and Disease Report 2023”, it is pointed out that the prevalence of cardiovascular diseases (CVD) in China continues to rise, it is estimated that the current number of people living with CVD in our country is 330 million, the mortality rate of CVD remains the highest (2). Diabetes and cardiovascular disease are closely related, with type 2 diabetes mellitus (T2DM) patients having a 2 to 4 times higher risk of developing CVD during their lifetime (3). Global Burden of Cardiovascular Diseases and Risk Factors (4) indicates that the prevalence of CVD among diabetic patients is as high as 31.5%, and CVD is the leading cause of death among diabetic patients in China (5). The most common complications among diabetic patients in China are hypertension (52.2%) and dyslipidemia (46.8%) (5), which further increase the risk of CVD. Comprehensive intervention targeting multiple cardiovascular risk factors, such as blood glucose, blood pressure, and blood lipids, can effectively reduce the incidence and mortality risk of CVD (6). Studies have shown that T2DM patients whose risk factors are controlled within target ranges have minimal risk of death, myocardial infarction, or stroke (7). Different countries and associations have proposed varying cardiovascular risk assessment tools and risk factor control targets for patients with T2DM, aiming to reduce the prevalence and mortality of CVD among T2DM patients. In August 2023, the European Society of Cardiology (ESC) released the “2023 ESC Guidelines for the management of cardiovascular disease in patients with diabetes.” For the first time, the guidelines introduced the SCORE2-Diabetes model (8), which stratifies the 10-year cardiovascular risk for patients with T2DM. The guidelines provide distinct targets for controlling cardiovascular risk factors and medication recommendations tailored to patients at different risk levels (9). Considering the substantial workload required to assess risk using the SCORE2-Diabetes model, its practical application in clinical settings poses challenges. The primary objective of this study is to compare the 10-year cardiovascular risk assessment methods, outlined in the SCORE2-Diabetes model, the “Comprehensive Type 2 Diabetes Management Algorithm - 2023 Update” released by the American Association of Clinical Endocrinologists (AACE) in 2023 (10) and the “Guideline for the Prevention and Treatment of Diabetes Mellitus in China (2024 Edition) “ issued by the Chinese Diabetes Society (CDS) (11). The aim is to identify a convenient and effective method for assessing 10-year cardiovascular risk in patients with T2DM for clinical use.

## Methods

### Study participants

Patients with T2DM hospitalized in the Second Affiliated Hospital of Hebei North University from August 2023 to October 2023. Inclusion criteria: (1) Aged between 40 and 69 years; (2) Complete examination indicators, including blood pressure, blood glucose, lipids, etc. Exclusion criteria: (1) Patients with diseases other than diabetes that can secondarily elevate blood glucose levels, such as hyperthyroidism/hypothyroidism, pancreatitis; (2) Patients who have recently used medications that affect body weight, blood pressure, blood glucose, and lipids, such as glucocorticoids or immunosuppressants; (3) Patients with malignant tumors; (4) Patients with cognitive impairments. The study was approved by the Ethics Committee of the Second Affiliated Hospital of Hebei North University. As the study involved the retrospective collection of medical record data and patient identities were anonymized, informed consent was waived.

### Data collection

This study is a retrospective analysis, and all information was sourced from the Hospital Information System. Information such as patients’ age, gender, smoking and drinking status, body mass index (BMI), creatinine levels, blood glucose, blood pressure, blood lipids, and other cardiovascular risk factors, as well as the presence of atherosclerotic cardiovascular disease (ASCVD), CKD and diabetic complications were collected. We also estimated the glomerular filtration rate (eGFR).

### Research methods

#### 1 Classify T2DM patients into risk categories according to the three aforementioned guidelines

The SCORE2-Diabetes model classification method from the European Society of Cardiology (hereinafter referred to as the “ESC method”): This methodology initially gathers information on patients’ CVD risk factors, including age, smoking status, blood pressure, total cholesterol, and high-density lipoprotein cholesterol levels, along with diabetes-specific parameters such as age at diagnosis, glycemic levels, and renal function. Subsequently, individual scores for each of these variables are identified using a cumulative scoring table. These individual scores are then aggregated to derive a total score. The total score is matched against the corresponding percentage in the risk table, adjusted according to the patient’s country of residence, which, based on incidence rates, is classified as a high-risk region for CVD in China. This percentage indicates the probability of the patient experiencing a cardiovascular event within the next 10 years.SCORE2-Diabetes < 5% is classified as low risk; 5% to < 10% as moderate risk; 10% to < 20% as high risk; and ≥ 20% or presence of ASCVD or severe target organ damage (TOD) is classified as very high risk.

The classification method in the “Comprehensive Type 2 Diabetes Management Algorithm - 2023 Update” from the American Association of Clinical Endocrinologists (hereinafter referred to as the “AACE method”):High risk (10-year risk <10%): Duration of T2DM <10 years, with <2 traditional ASCVD risk factors, and no target organ damage; Very high risk (10-year risk 10%-20%): Duration of T2DM >10 years, with 2 traditional ASCVD risk factors, and no target organ damage; Extreme risk (10-year risk >20%): T2DM or prediabetes combined with ASCVD or target organ damage (left ventricular systolic or diastolic dysfunction, eGFR < 45mL/min/1.73m², or ankle-brachial index [ABI] <0.9).The traditional ASCVD risk factors include: advanced age, hypertension, CKD stage ≥3, smoking, male < 55 years and female < 65 years with a family history of premature ASCVD, low high-density lipoprotein cholesterol (HDL-C), or high non-HDL-C.

The classification method in the “Guideline for the Prevention and Treatment of Diabetes Mellitus in China (2024 Edition) “ (hereinafter referred to as the “CDS method”):Very high risk: Diabetic patients with a definitive history of ASCVD; High risk: Diabetic patients aged ≥40 years, or 20–39 years with ≥3 risk factors or with target organ damage; Moderate risk: The remaining patients with T2DM.

#### 2 Assess the achievement of blood pressure, blood glucose, and blood lipid targets in T2DM patients across the aforementioned different risk categories

The criteria from the “2023 ESC Guidelines for the management of cardiovascular disease in patients with diabetes”, the “Comprehensive Management Process for Type 2 Diabetes (2023 Edition)”, and the “Guideline for the Prevention and Treatment of Diabetes Mellitus in China (2024 Edition) “ (hereinafter referred to as the “ESC criteria,” the “AACE criteria,” and the “CDS criteria” respectively) all require blood pressure to be less than 130 mmHg for systolic blood pressure and less than 80 mmHg for diastolic blood pressure. For the requirements on blood glucose: the “ESC criteria” sets the glycated hemoglobin (HbA1c) at <7%, the “AACE criteria” at HbA1c <6.5%, fasting plasma glucose <6.11 mmol/L, and 2-hour postprandial glucose <7.78 mmol/L, while the “CDS criteria” sets HbA1c at <7%, fasting plasma glucose at 4.4-7.0 mmol/L, and non-fasting plasma glucose at <10 mmol/L. The requirements for blood lipids are shown in **Table 1**.

**Table 1:** Lipid control targets for T2DM patients with different ASCVD risk levels.

Risk level	LDL-C (mmol/L)	non-HDL-C (mmol/L)	Apo B (g/L)
European Society of Cardiology			
Moderate risk (10-year risk 5%-10%)	<2.6	-	-
High risk (10-year risk 10%-20%)	<1.8	-	-
Very high risk (10-year risk ≥20%)	<1.4	-	-
American Association of Clinical Endocrinologists			
High risk (10-year risk <10%)	<2.59	<3.3592	<0.9
Very high risk (10-year risk 10%-20%)	<1.813	<2.584	<0.8
Extreme risk (10-year risk >20%)	<1.4245	<2.3256	<0.7
Chinese Diabetes Society			
High risk (diabetes patients aged ≥40 years without a history of ASCVD)	<1.8	<2.6	-
Very high risk (diabetes patients with a definitive history of ASCVD)	<1.4	<2.2	-

ASCVD, atherosclerotic cardiovascular disease; HDL-C, high-density lipoprotein cholesterol; LDL-C, low-density lipoprotein cholesterol; Apo B, apolipoprotein B.

### Statistical methods

Data were analyzed using SPSS, version 27. Continuous variables were tested for normality using the Kolmogorov-Smirnov test. Variables that followed a normal distribution were expressed as mean ± criteria deviation. T-tests were used for comparisons between groups, while ANOVA was used for comparisons among multiple groups, with Bonferroni correction or pairwise comparisons performed as necessary. Variables that did not follow a normal distribution were expressed as median and quartiles, and comparisons between groups were made using the rank-sum test. Categorical variables are expressed as percentages, and comparisons of rates among groups were conducted using chi-square (χ²) tests or Fisher’s exact test. For paired three-sample comparisons, the Friedman two-way analysis of variance by ranks was used. A P-value < 0.05 was considered statistically significant.

## Results

**Figure 1:**
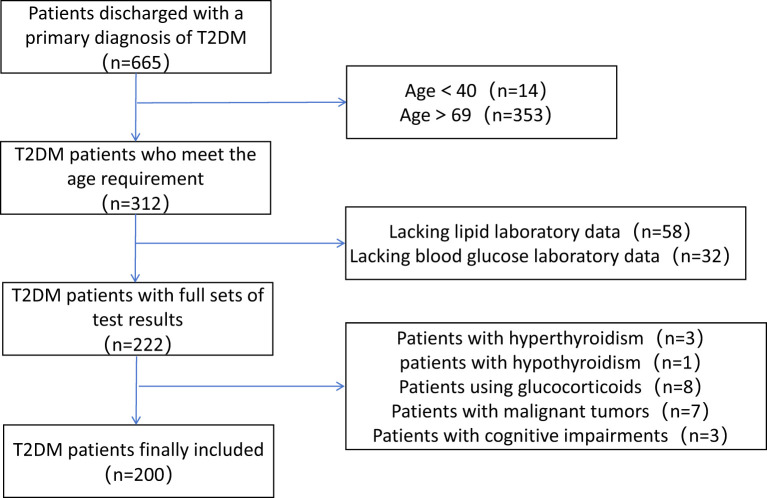
Flowchart of patient selection.

This study included a total of 200 hospitalized T2DM patients, the detailed patient selection process is shown in **Figure 1**. The baseline characteristics of the study subjects are presented in **Table 2**. The median age was 62.5 (57, 67), with 97 male patients. Females had higher levels of systolic blood pressure, 2-hour postprandial glucose, total cholesterol, LDL-C, non-HDL-C, apolipoprotein B, and HDL-C compared to males.

**Table 2:** Baseline characteristics of type 2 diabetes patients [n(%)].

Item	Total (n=200)	Men (n=97)	Women (n=103)	P value
Age (years)	62.5 (57,67)	62 (55,66)	63 (58,67)	0.176
Body Mass Index (kg/m^2^, $\bar{x}\pm s$ )	25.15 ± 3.22	25.05 ± 2.97	25.24 ± 3.45	0.670
Systolic Blood Pressure (mmHg, $\bar{x}\pm s$ )	138.50 ± 19.86	135.45 ± 18.81	141.37 ± 20.48	0.034
Diastolic Blood Pressure (mmHg, $\bar{x}\pm s$ )	84.30 ± 13.22	85.04 ± 13.42	83.61 ± 13.86	0.450
Hemoglobin A1c (%, $\bar{x}\pm s$ )	8.50 ± 1.90	8.32 ± 1.95	8.6 ± 1.85	0.180
Fasting Blood Glucose (mmol/L, $\bar{x}\pm s$ )	9.65 ± 3.72	9.43 ± 3.55	9.86 ± 3.88	0.420
Postprandial 2-hour Blood Glucose (mmol/L, $\bar{x}\pm s$ )	13.90 ± 5.07	13.12 ± 4.99	14.63 ± 5.05	0.034
Total Cholesterol (mmol/L, $\bar{x}\pm s$ )	4.45 ± 1.41	4.10 ± 1.26	4.78 ± 1.47	0.001
HDL-C (mmol/L, $\bar{x}\pm s$ )	1.04 ± 0.28	0.97 ± 0.25	1.10 ± 0.29	<0.001
LDL-C (mmol/L, $\bar{x}\pm s$ )	2.61 ± 0.88	2.46 ± 0.85	2.76 ± 0.90	0.017
non-HDL-C (mmol/L, $\bar{x}\pm s$ )	3.41 ± 1.31	3.13 ± 1.19	3.68 ± 1.36	0.003
Apolipoprotein B (g/L, $\bar{x}\pm s$ )	0.97 ± 0.28	0.91 ± 0.27	1.02 ± 0.29	0.006
Lipoprotein (a) (mg/L)	140 (55.20,301.80)	140.9 (60.55,300.2)	139.1 (54.00,308.00)	0.770
Glomerular Filtration Rate (mL/min/1.73m^2^, $\bar{x}\pm s$ )	90.36 ± 21.18	91.75 ± 19.39	89.05 ± 22.76	0.360
Smoking	43 (21.50)	42 (43.30)	1 (0.97)	<0.001
Drinking Alcohol	30 (15.00)	30 (30.93)	0 (0)	<0.001
Uses of lipid-lowering medication	34 (17.00)	21 (21.60)	13 (12.60)	0.089
Statins	33 (16.50)	20 (20.62)	13 (12.62)	0.128
Other lipid-lowering agents	3 (1.50)	3 (3.09)	0 (0)	0.224
Uses of glucose-lowering medication	147 (73.50)	71 (73.20)	76 (73.80)	0.925
Basal insulin	30 (15.00)	12 (12.37)	18 (17.48)	0.312
Premixed insulin	17 (8.50)	9 (9.30)	8 (7.80)	0.702
Prandial insulin	17 (8.50)	6 (6.20)	11 (10.68)	0.255
Metformin	103 (51.50)	48 (49.5)	55 (53.4)	0.580
SGLT2i	11 (5.50)	6 (6.20)	5 (4.90)	0.680
GLP-1 RA	1 (0.50)	0 (0)	1 (1.00)	1.000
Sulfonylureas	40 (20.00)	17 (17.50)	23 (22.30)	0.396
Glucosidase inhibitors	42 (21.00)	21 (21.60)	21 (20.40)	0.827
DPP-4i	6 (3.00)	4 (4.10)	2 (1.90)	0.625
Thiazolidinediones	8 (4.00)	5 (5.20)	3 (2.90)	0.654
Non-sulfonylurea insulin secretagogues	6 (3.00)	2 (2.10)	4 (3.90)	0.734
Uses of antihypertensive medication	95 (47.50)	46 (47.40)	49 (47.60)	0.983
ACEI	12 (6.00)	5 (5.20)	7 (6.80)	0.625
ARB	31 (15.50)	10 (10.30)	21 (20.4)	0.049
CCB	56 (28.00)	27 (27.80)	29 (28.20)	0.960
β-blockers	20 (10.00)	9 (9.30)	11 (10.70)	0.741
Diuretics	9 (4.50)	5 (5.20)	4 (3.90)	0.927
Other antihypertensive agents	10 (5.00)	6 (6.20)	4 (3.90)	0.673

HDL-C, high-density lipoprotein cholesterol; LDL-C, low-density lipoprotein cholesterol;SGLT2i, sodium-glucose co-transporter 2 inhibitor; GLP-1 RA, glucagon-like peptide-1 receptor agonist; DPP-4i, dipeptidyl peptidase-4 inhibitor; ACEI, angiotensin-converting enzyme inhibitor; ARB, angiotensin II receptor blocker; CCB, calcium channel blocker.

**Figure 2:**
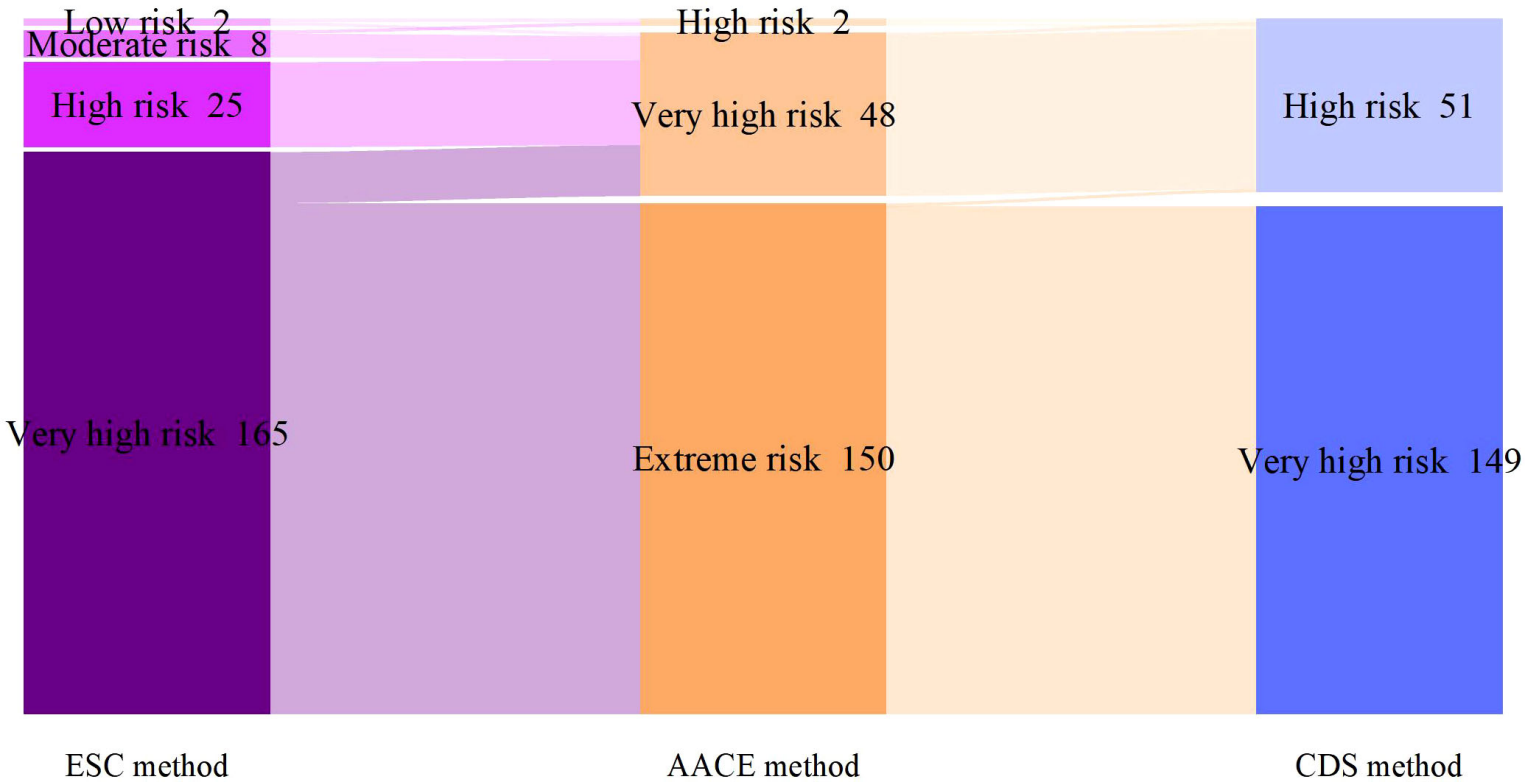
Comparison of cardiovascular risk stratification for all T2DM patients under different criteria.

**Figure 3:**
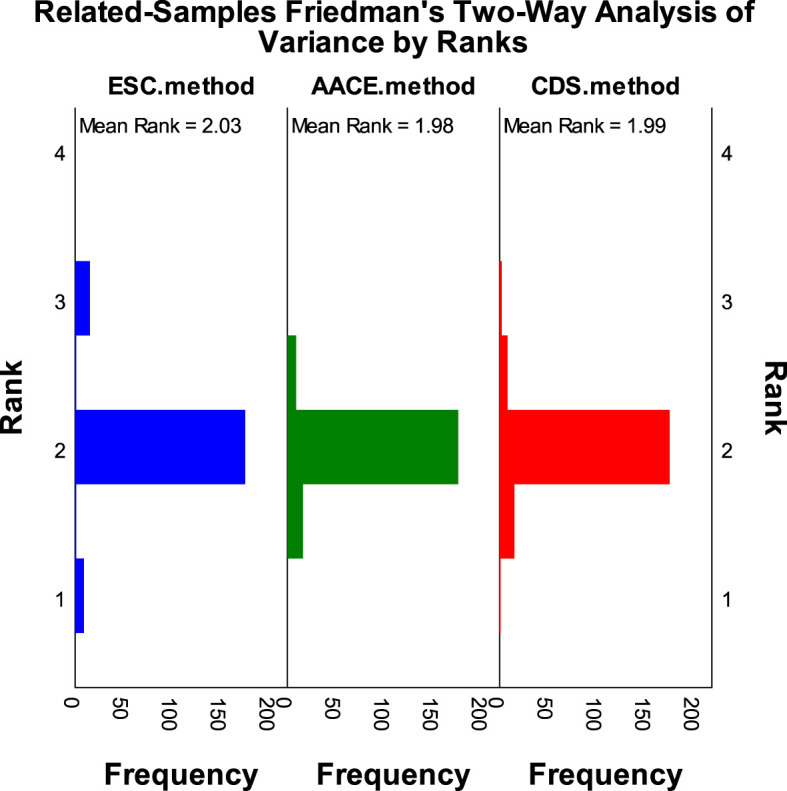
Rank variations of cardiovascular risk categories for all T2DM patients under different methods (Friedman method).

**Figure 4:**
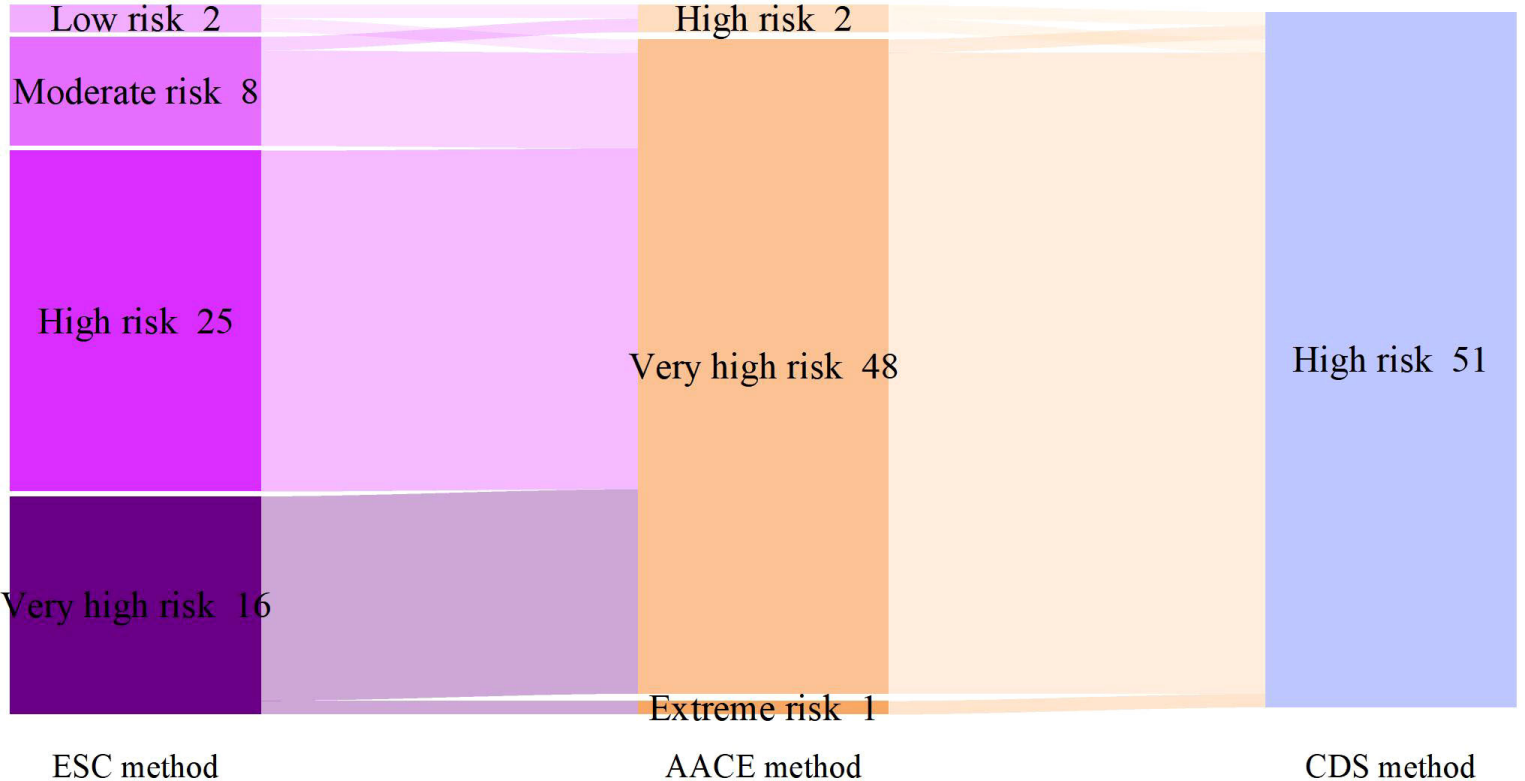
Comparison of cardiovascular risk stratification for T2DM patients without ASCVD under different criteria.

**Figure 5:**
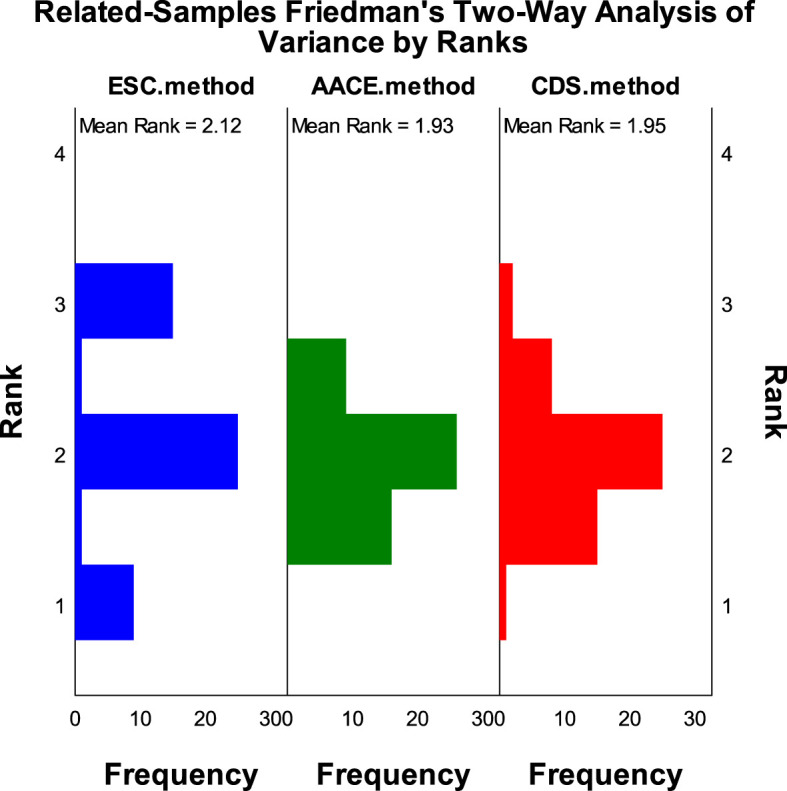
Rank variations of cardiovascular risk categories for T2DM patients without ASCVD under different methods (Friedman method).

According to the SCORE2-Diabetes model classification: 165 cases (82.5%) were classified as very high risk, 25 cases (10%) as high risk, 8 cases (4%) as moderate risk, and 2 cases (1%) as low risk; The results using the “AACE method” were as follows: 150 cases (75%) were classified as extreme risk, 48 cases (24%) as very high risk, and 2 cases (1%) as high risk; The results using the “CDS method” were as follows: 149 cases (74.5%) were classified as very high risk, and 51 cases (25.5%) as high risk (**Figure 2**). When comparing the results of risk category classification for T2DM patients among the three guidelines, there was no statistically significant difference (χ²=2.759, P=0.252, Friedman two-way analysis of variance, **Figure 3**). Considering that 149 patients were classified into the highest risk group due to having ASCVD, these patients were excluded from the analysis (**Figure 4**). Upon re-comparing the results of the risk category classification, there was still no statistically significant difference (χ²=2.759, P=0.252, Friedman two-way analysis of variance, **Figure 5**).

The achievement of risk factor targets and clinical characteristics of patients with type 2 diabetes based on the “ESC criteria” (**Table 3**). There were significant differences in the achievement rates of blood pressure and lipid targets among patients in the four risk categories, mainly reflected in the higher blood pressure achievement rates in moderate-risk patients compared to very high-risk patients (62.5% vs 19.4%), the achievement rates of lipid levels in moderate-risk patients was also higher than that in very high-risk patients (50% vs 6.1%), the achievement rates of systolic blood pressure in high-risk patients was higher than that in very high-risk patients (56% vs 31.5%); Regarding the levels of risk factors, the systolic blood pressure in moderate-risk patients (122 ± 20.34) was lower than that in very high-risk patients (140.28 ± 19.59), and all the above P-values were < 0.05.

**Table 3:** Achievement and levels of risk factor control among patients with T2DM according to ESC criteria [n (%)].

Item	Very high risk (n=165)	High risk (n=25)	Moderate risk (n=8)	Low risk (n=2)	P
Achievement of blood pressure targets	32 (19.4)	7 (28.0)	5 (62.5)^*^	1 (50.0)	0.016
Achievement of blood glucose targets	36 (21.8)	5 (20.0)	1 (12.5)	1 (50.0)	0.730
Achievement of blood lipid targets	10 (6.1)	3 (12.0)	4 (50.0)^*^	2 (100.0)	<0.001
Achievement of blood pressure, blood glucose, and lipid targets	0 (0.0)	0 (0.0)	1 (12.5)	1 (50.0)	0.001
Achievement of systolic blood pressure targets	52 (31.5)	14 (56.0)*	5 (62.5)	1 (50.0)	0.020
Achievement of diastolic blood pressure targets	54 (32.7)	9 (36.0)	5 (62.5)	1 (50.0)	0.295
Systolic Blood Pressure (mmHg, $\bar{x}\pm s$ )	140.28 ± 19.59	132.4 ± 19.29	122 ± 20.34^*^	133.5 ± 4.95	0.024
Diastolic Blood Pressure (mmHg, $\bar{x}\pm s$ )	84.88 ± 13.44	82.92 ± 11.96	78.25 ± 12.78	78.5 ± 12.02	0.452
Hemoglobin A1c (%, $\bar{x}\pm s$ )	8.48 ± 1.87	8.524 ± 1.60	9.35 ± 3.16	6.55 ± 0.78	0.295
LDL-C (mmol/L, $\bar{x}\pm s$ )	2.58 ± 0.88	2.86 ± 0.95	2.72 ± 0.75	1.79 ± 0.01	0.255

LDL-C, low-density lipoprotein cholesterol; * indicates P < 0.05 compared to the very high-risk group.

The achievement rates of risk factors and clinical characteristics of type 2 diabetes patients based on the “AACE criteria” (**Table 4**): The differences in risk factors among the three risk categories were mainly reflected in blood pressure, the achievement rates of systolic blood pressure in very high-risk patients was higher than that in extreme-risk patients (52.1% vs 30.7%),the mean systolic blood pressure in very high-risk patients (132.31 ± 19.28) was also lower than that in extreme-risk patients (140.61 ± 19.76), and all the above P-values were < 0.05.

**Table 4:** Achievement and levels of risk factor control among patients with T2DM according to AACE criteria [n (%)].

Item	Extreme risk (n=150)	Very high risk (n=48)	High risk (n=2)	P
Achievement of blood pressure targets	29 (19.3)	15 (31.3)	1 (50.0)	0.105
Achievement of blood glucose targets	2 (1.3)	2 (4.2)	0 (0.0)	0.278
Achievement of blood lipid targets	10 (6.7)	6 (12.5)	2 (100.0)	0.002
Achievement of blood pressure, blood glucose, and lipid targets	0 (0.0)	0 (0.0)	0 (0.0)	-
Achievement of systolic blood pressure targets	46 (30.7)	25 (52.1)^*^	1 (50.0)	0.010
Achievement of diastolic blood pressure targets	49 (32.7)	19 (39.6)	1 (50.0)	0.460
Achievement of HbA1c targets	13 (8.7)	5 (10.4)	0 (0.0)	0.813
Achievement of fasting blood glucose targets	17 (11.3)	4 (8.3)	1 (50.0)	0.255
Achievement of 2-hour postprandial blood glucose targets	10 (6.7)	7 (14.6)	0 (0.0)	0.275
Achievement of LDL-C targets	11 (7.3)	6 (12.5)	2 (100.0)	0.004
Achievement of non-HDL-C targets	30 (20.0)	9 (18.8)	2 (100.0)	0.068
Achievement of apolipoprotein B targets	26 (17.3)	10 (20.8)	2 (100.0)	0.036
Systolic Blood Pressure (mmHg, $\bar{x}\pm s$ )	140.61 ± 19.76	132.31 ± 19.28^*^	128.5 ± 12.02	0.032
Diastolic Blood Pressure (mmHg, $\bar{x}\pm s$ )	85.23 ± 13.67	81.54 ± 111.65	81.5 ± 7.78	0.234
Hemoglobin A1c (%, $\bar{x}\pm s$ )	8.48 ± 1.90	8.61 ± 1.96	7.55 ± 0.64	0.712
Fasting Blood Glucose (mmol/L, $\bar{x}\pm s$ )	9.55 ± 3.68	10.11 ± 3.83	6.63 ± 3.17	0.341
Postprandial 2-hour Blood Glucose (mmol/L, $\bar{x}\pm s$ )	13.95 ± 4.96	13.82 ± 5.52	12.05 ± 0.07	0.866
LDL-C (mmol/L, $\bar{x}\pm s$ )	2.57 ± 0.89	2.78 ± 0.86	1.77 ± 0.01	0.136
non-HDL-C (mmol/L, $\bar{x}\pm s$ )	3.38 ± 1.30	3.57 ± 1.35	2.28 ± 0.07	0.329
Apolipoprotein B (g/L, $\bar{x}\pm s$ )	0.96 ± 0.29	1.01 ± 0.27	0.68 ± 0.01	0.200

LDL-C, low-density lipoprotein cholesterol; non-HDL-C, non-high-density lipoprotein cholesterol; * indicates P < 0.05 compared to the extreme risk.

achievement rates of risk factors and clinical characteristics of type 2 diabetes patients based on the “CDS criteria” (**Table 5**): The achievement rates of systolic blood pressure in high-risk patients was higher than that in very high-risk patients (51% vs 30%). The mean systolic blood pressure level in high-risk patients (132.71 ± 19.19) was also lower than that in very high-risk patients (140.48 ± 19.76), and all the above P-values were < 0.05.

**Table 5:** Achievement and levels of risk factor control among patients with T2DM according to CDS criteria [n (%)].

Item	Very high risk (n=149)	High risk (n=51)	P
Achievement of blood pressure targets	29 (19.5)	16 (31.4)	0.079
Achievement of blood glucose targets	13 (8.7)	6 (11.8)	0.717
Achievement of blood lipid targets	10 (6.7)	8 (15.7)	0.099
Achievement of blood pressure, blood glucose, and lipid targets	0 (0.0)	0 (0.0)	-
Achievement of systolic blood pressure targets	46 (30.9)	26 (51.0)	0.010
Achievement of diastolic blood pressure targets	49 (32.9)	20 (39.2)	0.412
Achievement of HbA1c targets	33 (22.1)	10 (19.6)	0.703
Achievement of fasting blood glucose targets	39 (26.2)	11 (21.6)	0.512
Achievement of non-fasting blood glucose targets	36 (24.2)	13 (25.5)	0.849
Achievement of LDL-C targets	10 (6.7)	8 (15.7)	0.099
Achievement of non-HDL-C targets	27 (18.1)	11 (21.6)	0.588
Systolic Blood Pressure (mmHg, $\bar{x}\pm s$ )	140.48 ± 19.76	132.71 ± 19.19	0.015
Diastolic Blood Pressure (mmHg, $\bar{x}\pm s$ )	85.11 ± 13.64	81.96 ± 11.74	0.143
Hemoglobin A1c (%, $\bar{x}\pm s$ )	8.45 ± 1.88	8.64 ± 1.98	0.550
Fasting Blood Glucose (mmol/L, $\bar{x}\pm s$ )	9.51 ± 3.67	10.07 ± 3.87	0.357
non-Fasting Blood Glucose (mmol/L, $\bar{x}\pm s$ )	13.88 ± 4.91	13.95 ± 5.55	0.932
LDL-C (mmol/L, $\bar{x}\pm s$ )	2.55 ± 0.86	2.80 ± 0.94	0.082
non-HDL-C (mmol/L, $\bar{x}\pm s$ )	3.34 ± 1.21	3.63 ± 1.56	0.177

LDL-C, low-density lipoprotein cholesterol; non-HDL-C, non-high-density lipoprotein cholesterol.

Comparison of the achievement rates of blood pressure, blood glucose, and lipids according to the three criteria (**Table 6**): Due to the consistent criteria for blood pressure, there was no difference in the achievement rates of blood pressure, which was 22.5% across all criteria; There are differences in blood glucose target achievement rates among the three criteria (P<0.001), while there is no statistically significant difference in lipid target achievement and overall target achievement. Under the “ESC criteria,” “AACE criteria,” and “CDS criteria,” the proportions of T2DM patients who achieved target levels for all three major cardiovascular risk factors were 1%, 0%, and 0%.

**Table 6:** Achievement rates of cardiovascular risk factors under different criteria [n (%)].

Item	ESC Criteria	AACE Criteria	CDS Criteria	P
Achievement of blood pressure targets	45 (22.5)	45 (22.5)	45 (22.5)	1.000
Achievement of blood glucose targets	43 (21.5)	4 (2.0)	19 (9.5)	<0.001
Achievement of blood lipid targets	19 (9.5)	18 (9.0)	18 (9.0)	0.980
Achievement of blood pressure, blood glucose, and lipid targets	2 (1.0)	0 (0.0)	0 (0.0)	0.332

## Discussion

The European Society of Cardiology recommends the use of the SCORE2-Diabetes model for assessing 10-year cardiovascular risk in patients with T2DM and proposes different lipid control targets for patients at different risk levels. However, the SCORE2-Diabetes model is relatively complex to operate and not convenient for use in clinical practice. Is there any difference between its assessment results and those of simpler methods for assessing 10-year cardiovascular risk in T2DM patients? Additionally, this model was developed from European populations, can it be applied to Asian populations? Based on this, we compared the SCORE2-Diabetes model from the ESC’s “Guidelines on Cardiovascular Disease Management in Diabetes (2023 version)”, the method in the AACE’s “Comprehensive Type 2 Diabetes Management Algorithm - 2023 Update”, and the method in the CDS’s “Guideline for the Prevention and Treatment of Diabetes Mellitus in China (2024 Edition)” for assessing 10-year cardiovascular risk in patients with type 2 diabetes. The results showed no statistically significant difference in the assessment outcomes between the SCORE2-Diabetes model and the “AACE method” and “CDS method” (χ²=2.759, P=0.252).The SCORE2-Diabetes model classified 165 (82.5%) patients into the very high-risk category, among which 149 patients were directly included due to having ASCVD without using the scoring chart. Considering the significant number of these patients, which might influence the results, we excluded them and re-compared the cardiovascular risk for the remaining 51 patients. The results still showed no statistically significant difference (χ²=2.759, P=0.252).A 3B study (12) conducted in China showed that when assessing the 10-year cardiovascular risk in type 2 diabetes patients using the 2019 ESC guidelines, the results categorized patients as very high risk (65.6%), high risk (7.5%), and moderate risk (0.6%).The remaining 26.4% could not be classified due to a shorter duration of T2DM and the presence of only 1–2 risk factors, thus falling into an indeterminate risk category. The results of the assessment using the three methods in this study are generally consistent with those of the 3B study.

Apart from the differences in assessment methods, the control targets for blood glucose and blood lipids also vary among the three guidelines. Therefore, we further compared the achievement rates of blood pressure, blood glucose, blood lipids, and the three major cardiovascular risk factors, among patients at different risk levels under different criteria. The results showed that the proportions of patients achieving target levels for all three risk factors (blood pressure, blood glucose, and blood lipids) were 1%, 0%, and 0% under the “ESC criteria”, “AACE criteria” and “CDS criteria” respectively, with no statistically significant difference in the achievement rates (P=0.332).A national cross-sectional survey study (13) showed that the proportion of patients meeting all three risk factor targets was 4.4%.The study results by Xiaoyun Yang (14) showed that in 2017, the proportion of patients who met the Chinese Diabetes Society’s targets (HbA1c < 7%, blood pressure < 130/80 mmHg, and normal lipid levels) was 0.9%.The primary reason for the aforementioned differences is the variation in study populations. Both our study and Xiaoyun Yang’s study focused on hospitalized T2DM patients, whose conditions are relatively more severe. Furthermore, this finding underscore the importance of focusing on the management of cardiovascular risk factors in T2DM patients to reduce CVD-related mortality.

In the analysis of blood pressure control, although the guidelines suggest that the target blood pressure values can be appropriately relaxed for elderly diabetic patients or those with severe coronary heart disease (9–11), due to the retrospective nature of this study and the difficulty in assessing patients’ physical conditions at the time of admission, the uniform target for blood pressure was set at <130/80 mmHg. The achievement rates for blood pressure was 22.5%, which is consistent with the findings of Xiaoyun Yang’s study (14) where the blood pressure control rate for T2DM patients in 2016 was 21.7%, as well as the blood pressure control rate of 22.2% reported in the nationwide cross-sectional survey study (13).There is no difference in blood pressure control between hospitalized and non-hospitalized patients. The reason for this may be that patients have a relatively clear perception of their blood pressure, which is also easy to monitor and control. Therefore, regardless of the severity of the illness, patients’ control over their blood pressure is consistent.

In the assessment results of the three criteria, the mean systolic blood pressure of patients at different risk levels showed statistically significant differences, indicating that blood pressure has a considerable impact on the 10-year cardiovascular risk level in T2DM patients. In contrast, no such differences were observed for blood glucose and lipids.

In the analysis of blood glucose achievement, the rates of achieving glycemic targets under the “ESC criteria,” “AACE criteria,” and “CDS criteria” were 21.5%, 2%, and 9.5%, respectively, with statistically significant differences (P<0.001). The achievement rates for HbA1c targets were 21.5%, 9%, and 21.5%, also showing statistically significant differences (P<0.001).The achievement rates for HbA1c was lower than that reported by Xu Minwei (15), whose study found an HbA1c achievement rates of 39.39%. The primary reason for this difference is that their study included T2DM patients managed within a regional health system, rather than hospitalized patients. Another national cross-sectional survey study (13) reported an HbA1c achievement rates of 64.1%, primarily because this study relaxed the HbA1c target to 8% for some elderly patients with complications. The differences in blood glucose targets across various criteria are considerable. This discrepancy may be influenced by fasting blood glucose and 2-hour postprandial blood glucose levels. Given that these two measures can vary significantly, we excluded them and analyzed only HbA1c, yet differences still persisted. The reason for this difference is that the “AACE criteria” require an HbA1c of <6.5%, which is stricter than the <7% required by the other two criteria. This indicates that there are significant differences in blood glucose control targets among the three criteria. Notably, the relatively high blood glucose treatment rate (73.5%) contrasts sharply with the low glycemic control rate (only 21.5%), highlighting a significant discrepancy between treatment initiation and effective diabetes management. This finding suggests that while a substantial proportion of patients are receiving therapy, the quality or adherence to treatment may be suboptimal. Several factors could contribute to this gap, including inadequate patient education, insufficient follow-up, or challenges in lifestyle modification. Therefore, improving glycemic outcomes will likely require a multifaceted approach involving both healthcare providers and patients, such as enhanced patient counseling, individualized treatment plans, and improved monitoring strategies.

In the analysis of lipid achievement, the rates of achieving lipid targets under the “ESC criteria,” “AACE criteria,” and “CDS criteria” were 9.5%, 9%, and 9%, respectively, with no statistically significant difference (P=0.980).A national cross-sectional survey study (13) showed that the achievement rates for lipid targets (LDL-C<1.8 mmol/L) was 23.9%. The differences in these rates can be attributed not only to variations in study populations but also to the more stringent lipid targets set by the three criteria, with the lowest LDL-C target being 1.4 mmol/L. Additionally, the “AACE criteria” and “CDS criteria” include requirements for non-HDL-C, which contributes to this discrepancy. In addition, the low achievement rate of lipid control is closely related to the low treatment rate among patients. As shown in **Table 2**, only 17% of patients received lipid-lowering therapy, indicating a significant gap in the management of dyslipidemia. This suggests that greater attention should be paid to lipid-lowering interventions in the future clinical management of these patients.

We conducted multiple group comparisons for the achievement rates and levels of risk factors across different risk categories under the “ESC criteria” and “AACE criteria.” For those risk factors where the comparison results showed statistically significant differences, we performed pairwise comparisons with Bonferroni correction. However, during this process, we excluded the low-risk group under the “ESC criteria” and the high-risk group under the “AACE criteria,” primarily due to the small number of individuals in these two groups, each comprising only 2 people.

In the baseline characteristics of the study participants, females had higher levels of systolic blood pressure, total cholesterol, LDL-C, and HDL-C compared to males (P<0.05), which is consistent with the findings of Zhang Min (16). This characteristic was also reflected in a Chinese study that included 37,317 participants (17), where the baseline lipid profiles similarly demonstrated this feature.

Limitations of this study: First, the study population consisted of hospitalized T2DM patients, who generally have more severe conditions compared to outpatient patients. Therefore, the overall control of risk factors was not ideal, and the 10-year cardiovascular risk was higher. Second, this study included data from only one tertiary hospital, where patients may have more severe conditions and therefore a higher cardiovascular risk profile. In addition, due to differences in health insurance reimbursement policies, patients with milder T2DM may prefer to be hospitalized in secondary hospitals, where they can benefit from higher reimbursement rates. This selection bias may affect the generalizability of our findings. Third, as this study was a preliminary and exploratory analysis, the number of collected cases is currently limited due to constraints in time and available human resources. To understand the 10-year cardiovascular risk profile of all T2DM patients, large-scale prospective clinical studies involving the entire T2DM population are still needed. Building on the current findings, we plan to conduct more comprehensive studies with larger cohorts and improved designs in the future.

This study was primarily conducted by clinical pharmacists specializing in cardiology. Future research aims to investigate the role of clinical pharmacists in reducing cardiovascular disease risk by following up with patients, providing medication guidance, and intervening in the control of risk factors. This approach seeks to address the shortage of clinical physicians, reduce the cardiovascular disease risk in T2DM patients, and highlight the value of clinical pharmacists (18, 19).

## Conclusion

Although the 10-year cardiovascular risk assessment methods and the criteria for achieving primary cardiovascular risk factors differ across the three guidelines, the assessment results and achievement rates for 200 T2DM patients showed no statistically significant differences.

## Data Availability

The raw data supporting the conclusions of this article will be made available by the authors, without undue reservation.
